# Semi-field evaluation of the exposure-free mosquito electrocuting trap and BG-Sentinel trap as an alternative to the human landing catch for measuring the efficacy of transfluthrin emanators against *Aedes aegypti*

**DOI:** 10.1186/s13071-021-04754-x

**Published:** 2021-05-20

**Authors:** Mgeni M. Tambwe, Adam Saddler, Ummi Abdul Kibondo, Rajabu Mashauri, Katharina S. Kreppel, Nicodem J. Govella, Sarah J. Moore

**Affiliations:** 1 Vector Control Product Testing Unit, Ifakara Health Institute, Environmental Health and Ecological Sciences, P.O. Box 74, Bagamoyo, Tanzania; 2 Swiss Tropical & Public Health Institute, Socinstrasse 57, 4051 Basel, Switzerland; 3 University of Basel, Petersplatz 1, 4001 Basel, Switzerland; 4 Telethon Kids Institute, Perth, Australia; 5 Nelson Mandela African Institution of Science and Technology (NM-AIST), P.O. Box 447, Tengeru, Tanzania; 6 Institute of Biodiversity, Animal Health and Comparative Medicine, University of Glasgow, Graham Kerr Building, Glasgow, G12 8QQ UK

**Keywords:** Mosquito electrocuting trap, Human landing catch, BG-Sentinel, Spatial repellent, *Ae. aegypti*

## Abstract

**Background:**

The human landing catch (HLC) measures human exposure to mosquito bites and evaluates the efficacy of vector control tools. However, it may expose volunteers to potentially infected mosquitoes. The mosquito electrocuting trap (MET) and BG-Sentinel traps (BGS) represent alternative, exposure-free methods for sampling host-seeking mosquitoes. This study investigates whether these methods can be effectively used as alternatives to HLC for measuring the efficacy of transfluthrin emanator against *Aedes aegypti.*

**Methods:**

The protective efficacy (PE) of freestanding passive transfluthrin emanators (FTPEs), measured by HLC, MET and BGS, was compared in no-choice and choice tests. The collection methods were conducted 2 m from an experimental hut with FTPEs positioned at 3 m on either side of them. For the choice experiment, a competitor HLC was included 10 m from the first collection point. One hundred laboratory-reared *Ae. aegypti* mosquitoes were released and collected for 3 consecutive h.

**Results:**

In the no-choice test, each method measured similar PE: HLC: 66% (95% confidence interval [CI]: 50–82), MET: 55% (95% CI: 48–63) and BGS: 64% (95% CI: 54–73). The proportion of mosquitoes recaptured was consistent between methods (20–24%) in treatment and varied (47–71%) in the control. However, in choice tests, the PE measured by each method varied: HLC: 37% (95% CI: 25–50%), MET: 76% (95% CI: 61–92) and BGS trap: 0% (95% CI: 0–100). Recaptured mosquitoes were no longer consistent between methods in treatment (2–26%) and remained variable in the control (7–42%). FTPE provided 50% PE to the second HLC 10 m away. In the control, the MET and the BGS were less efficacious in collecting mosquitoes in the presence of a second HLC.

**Conclusions:**

Measuring the PE in isolation was fairly consistent for HLC, MET and BGS. Because HLC is not advisable, it is reasonable to use either MET or BGS as a proxy for HLC for testing volatile pyrethroid (VP) in areas of active arbovirus-endemic areas. The presence of a human host in close proximity invalidated the PE estimates from BGS and METs. Findings also indicated that transfluthrin can protect multiple people in the peridomestic area and that at short range mosquitoes select humans over the BGS.

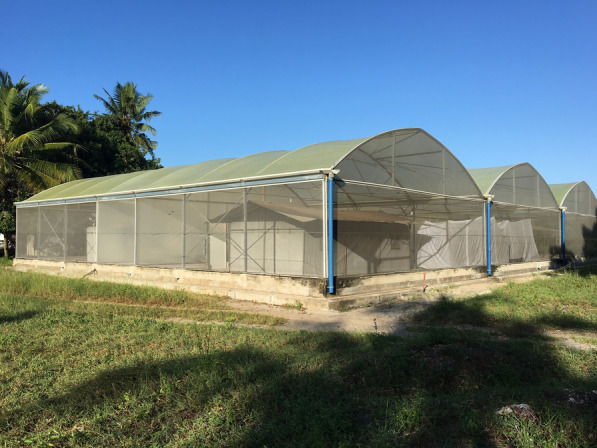

**Supplementary Information:**

The online version contains supplementary material available at 10.1186/s13071-021-04754-x.

## Background

*Aedes aegypti* (*Ae. aegypti*) and *Aedes albopictus* (*Ae. albopictus*) mosquitoes are responsible for the transmission of human arboviruses including dengue, yellow fever, chikungunya and zika viruses [1–4]. These mosquitos are well adapted to living in urban areas and bite during the daytime. The main vector control strategies deployed against *Aedes* vectors are larval source reduction, indoor residual spraying and space spraying [4, 5]. However, these control tools are labour intensive, costly and difficult to implement considering that *Ae. aegypti* mosquitoes can breed or rest in a wide range of small, transient and often cryptic places [4]. While these vector control approaches are useful, simple and more cost-effective control strategies against *Ae. aegypti* mosquitoes are urgently needed because of the increased frequency of epidemics and the geographical spread of a number of arboviruses [6]. Promising new strategies, including oviposition traps, transgenic mosquitoes, volatile pyrethroids (VP) and the use of *Wolbachia* spp., are currently under evaluation [7–9].

The impact of new vector control strategies is measured through entomological indicators, including vector density and human exposure to mosquito bites [10–12], which have often been measured by human landing catch (HLC) [13]. Human landing catch is the gold standard measure of human-vector exposure whereby, using an aspirator, human volunteers collect host-seeking mosquitoes that land on the volunteers’ exposed legs [14]. The numbers of mosquitoes caught (the human landing rate) approximate the number of mosquitoes that would bite one person at a particular time and place [10, 15]. This is a simple method and a direct measure of human-vector contact for both indoor- and outdoor-biting mosquitoes.

However, ethical and technical concerns arise when HLC is performed in disease-endemic areas. Because no prophylaxis or vaccine is available for most arboviral diseases, with the exception of yellow fever, putting the catcher at risk of contracting an arboviral disease [16]. Furthermore, differences in skills and motivation of the collectors may also introduce variation into the collected data. HLC is often performed over several hours, so the quality of data obtained may decline over time as the collectors tire or lose concentration. These technical drawbacks can be countered by proper training and supervision of the collectors, but are unlikely to be eliminated. Thus, it might be difficult to standardize data collected through this method by different research institutions.

Previous studies have established that mosquito traps baited with odor lures that mimic human chemical attractants have the potential to be used as an alternative to HLC for sampling mosquitoes [17–20]. Estimating human-mosquito contact accurately is vital for studies aiming to determine the disease risk of a certain area by calculating the entomological inoculation rate. Even if traps do not reflect the exact number of mosquitoes caught by HLC, for those that consistently catch less or more than HLC, correction factors can be used to obtain estimated counts. Similarly, for the testing of vector control tools, traps do not necessarily have to catch exactly the same number of mosquitoes as HLC; however, it is vital that they accurately reflect the impact of the vector control intervention. Several odor-baited traps have been demonstrated to be an appropriate alternative to HLC for measuring mosquito densities of various species such as *Anopheles* and *Aedes* [18, 19]. Knowledge of whether the presence in the environment of behavior-modifying compounds such as repellents affect the relative efficacy of odor-baited lures is limited [21]. If, as in the case of Okumu et al. [21], the repellent increases the attractiveness of the odor lure, then it will give an inaccurate picture of the efficacy of the repellent in reducing human-vector contact.

Furthermore, when traps are used in the field, competing sources of host odor are present. It is therefore important to determine whether mosquitoes may be diverted from traps to other hosts and whether this diversion is exacerbated by the presence of a spatial repellent. It is not unforeseeable that if an odor lure is already weaker than a human at attracting mosquitoes [22], then introducing a spatial repellent will mean that some host seeking mosquitoes will go toward the stronger pull of the human. If this is the case, then the traps using odor lures may overestimate the efficacy of the spatial repellent. Traps with a human lure, such as the MET or the human-baited double net trap, may therefore provide a more accurate measure of the efficacy of a spatial repellent [18, 23, 24].

While an enormous body of knowledge is available on the comparison of trap efficiency, no information is available on whether exposure-free methods (METs and the BGS trap) are suitable for testing spatial repellents such as volatile pyrethroids (VP). Therefore, the present study investigates three trapping methods—HLC, BGS and MET—for their ability to measure the protection provided by the VP transfluthrin against *Ae. aegypti* mosquitoes and whether an alternative host can affect this protection. Two experiments were performed: (i) a no-choice experiment in which protective efficacy (PE) was measured with the traps used in isolation and (ii) a choice test in which protective efficacy was measured with the traps used in the presence of a HLC.

## Methods

### Study site

**Fig. 1:**
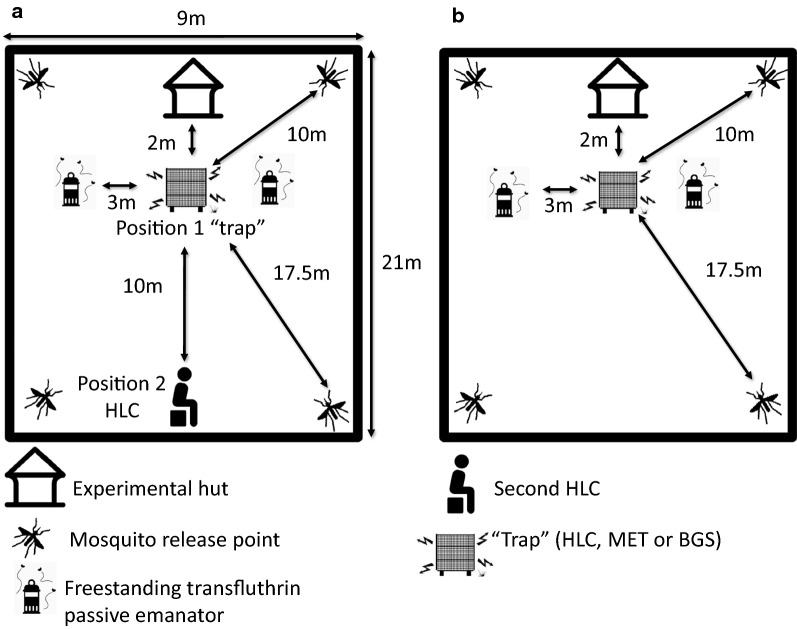
Experimental setup in the SFS. **a** Schematic representation of the choice experiment with the HLC competitor. **b** Schematic representation of the no-choice experiment without the HLC competitor. To mimic outdoor conditions, the experiment was conducted outside the experimental hut fixed inside the compartment of the semi-field system. Shown at the corners are mosquito release points

The experiment was conducted in the semi-field system (SFS) located in Bagamoyo, Tanzania, from January to June 2019. The SFS consists of large screened compartments that allow controlled experiments with disease-free laboratory-reared mosquitoes to be safely conducted under ambient climatic conditions [25]. Experiments can be replicated within a short period of time by releasing the same number of laboratory-reared mosquitoes each time without bias introduced by the natural daily heterogeneity in mosquito numbers that normally occurs in the field. The SFS is divided into two equal compartments, each measuring 9 m × 21 m (Fig. 1a and b), which were used for the experiment with a middle corridor acting as a buffer. The walls of the middle corridor are made from heavy-duty polyethylene, thus preventing airflow between the chambers. This allowed the independent evaluation of the traps in the presence or absence of a spatial repellent to be conducted simultaneously. The mean temperature and relative humidity were 24 °C and 83%, respectively.

### Mosquitoes

The experiments used laboratory-reared *Ae. aegypti* mosquitoes (Bagamoyo strain) originally colonized from Bagamoyo, Tanzania, and maintained at the Bagamoyo branch of the Ifakara Health Institute (IHI) since December 2015. The mosquitoes are susceptible to all classes of insecticides. The colony larvae were fed on Tropical fish flakes® until pupation, after which they were transferred to emergence bowls inside a 30 cm × 30 cm × 30 cm cage. The adult colony was fed on glucose 10% ad libitum, and cattle blood meals were given to adult females using direct membrane feeding assay for egg production on days 3, 6 and 9. The colony is maintained approximately at 12:12 (light:dark) natural light, 27 ± 2 °C and 80 ± 20% relative humidity.

For the purpose of this experiment, 3- to 8-day-old nulliparous female mosquitoes were used. Three cages of mosquitoes were sugar-starved for 12 h, then two hours before the experiment started, active probing female mosquitoes were selected from the cages to the small cages to ensure that only avid and fit mosquitoes were used. The mosquitoes were transferred to the SFS in smaller holding cages (10 cm × 10 cm × 10 cm with 25 mosquitoes each).

### Collection methods

#### Human landing catches

**Fig. 2:**
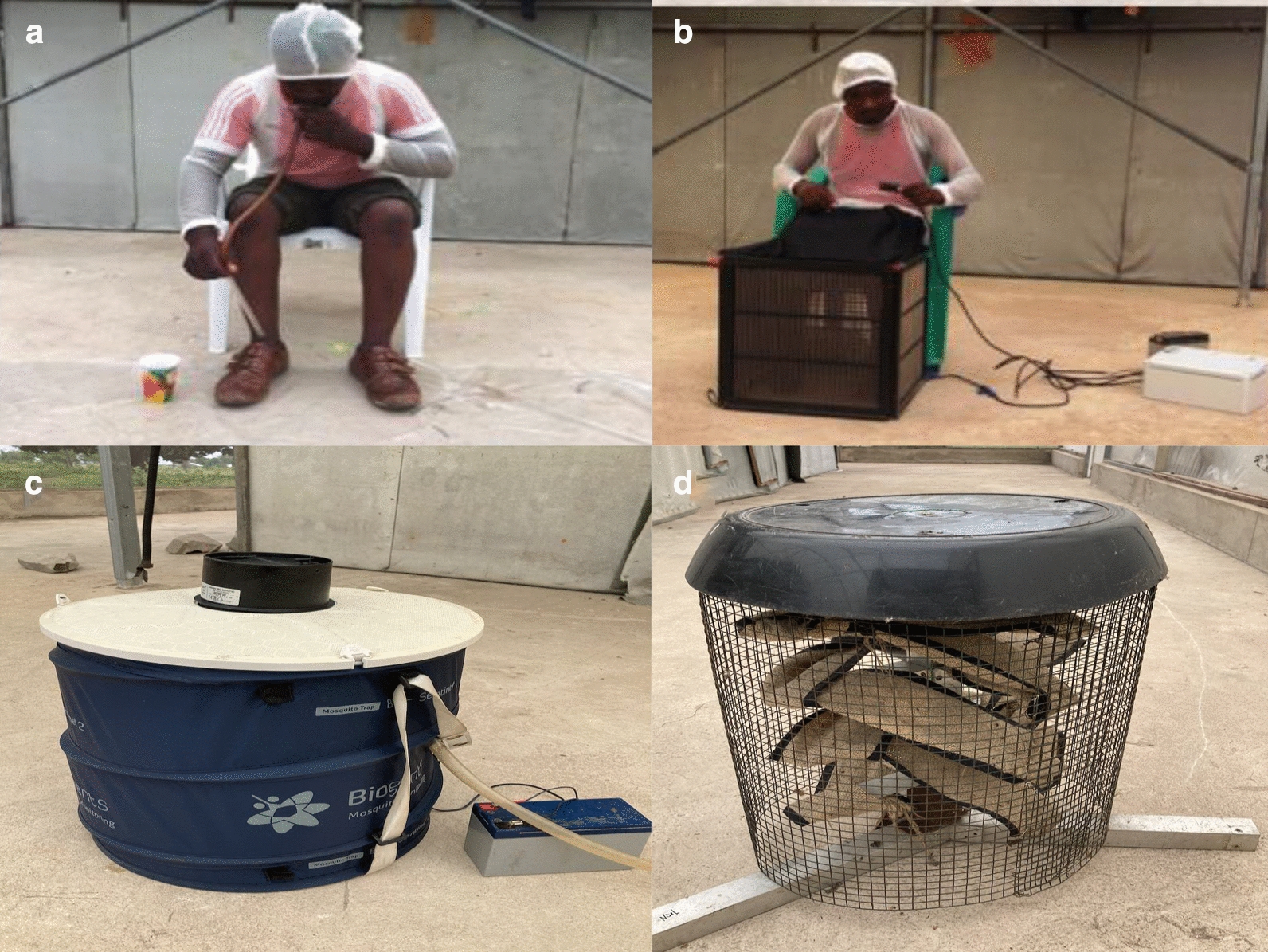
Collection methods and FTPE used in the study. **a** A volunteer conducting HLC. **b** A volunteer sitting on the chair with his leg inserted in an MET. **c** A BG-Sentinel trap with the battery and silicon tube supplying CO_2_. **d** An FTPE device as a source of transfluthrin

Four male volunteers aged 25–35 years, experienced in conducting HLC, were recruited upon informed consent. Because observation in previous experiments showed that *Ae. aegypti* bite all over the body, volunteers were covered with net jackets to prevent bites on areas where HLC was not taking place (Fig. 2a). For HLC, the volunteers sat on chairs exposing their legs between the ankle and knee (Fig. 2a), aspirated any mosquito that landed, and gently expelled them into a paper cup.

#### Mosquito-electrocuting trap (MET)

Previous experiments have demonstrated that METs could be used for sampling *Anopheles* mosquitoes [23] and *Aedes* mosquitoes [26]. The MET consists of an electric grid and a power-supply box. The electric grid is made up of four panels, each measuring 30 cm × 30 cm, in a square frame (Fig. 2b). Volunteers (the same as those recruited for HLC) put their legs within the frame in a similar fashion as for HLC, and host-seeking mosquitoes approaching the volunteers are intercepted and killed on their attempt to pass through the electrified grids before landing on a human. In this way, volunteers are protected from mosquito bites and, consequently, from exposure to mosquito-borne infection. Preliminary testing of optimal voltage for electrocution of *Ae. aegypti* identified that 680 V is sufficient to kill mosquitoes (with the specimen remaining intact) without causing harm in accidental contact with the volunteer. The trap is designed such that electrocution occurs when a mosquito touches the two parallel wires of the electric grid [24].

#### BG-Sentinel (BGS) trap

The BGS trap (Biogents AG, Regensburg, Germany) has been widely used as the standard trap for collection of adult *Aedes* mosquitoes [10, 22]. It is used together with the BG-Lure (Fig. 2c), a synthetic lure consisting of lactic acid, caproic acid and ammonium bicarbonate dispensed via granules in the specified channel [22]. Despite the lure’s being effective for 5 months, a new lure was used for each experimental round of 8 days. Carbon dioxide was released from a pressurized cylinder at the rate of 500 ml/min, using an acrylic gas flow meter (Hangzhou Darhor Technology Co., Limited, China). The operation of the BGS trap has been explained elsewhere [27, 28].

### Preparation of the freestanding transfluthrin passive emanator (FTPE)

Previous work showed that transfluthrin freestanding passive emanators (FTPEs) used under simulated outdoor conditions could significantly reduce the human landing rate of *Ae. aegypti* [29]. This device is a stool-like structure that supports hessian strips (made from plants of the species *Corchorus olitorius* or *C. capsularis*, also called jute, burlap or gunnysacks) (Fig. 2d). The hessian strips were made from hessian sacks purchased locally, washed using well water and powder detergent (OMO**®**, Unilever, Nairobi, Kenya) and dried under direct sunlight. They were then cut into strips measuring 5 m × 10 cm and treated with 5.25 g of transfluthrin emulsified concentrate (EC; Bayothrin EC, Bayer AG, Monheim am Rhein, Germany). Two FTPEs with a total of 10.50 g (5.25 g each) of transfluthrin were used per experiment.

### Experimental procedure

Experiments were conducted to compare the protective efficacies of the HLC, MET and BGS traps under no-choice (traps alone) and choice (with additional HLC) conditions.

In the choice assay (Fig. 1), one type of sampling trap was allocated between the two chambers of the SFS with one as treatment and the other as control, and experimentation was conducted for 8 consecutive days before switching to another type of trap. Once the treated and untreated emanators had been assigned to a particular chamber (treatment or control), they remained there for 4 consecutive experimental days and were then exchanged between the chambers to minimize the potential bias between chambers which could arise due to variation in wind direction. In the treatment chamber, two treated FTPEs were placed 3 m apart side by side with the trapping method while in the control, a similar fashion of placement of untreated FTPEs around the trap was employed (Fig. 1a, b). Two volunteers exchanged positions between the two chambers after every experimental day to account for potential bias due to differential attractiveness to mosquitoes between individuals [30].

The collection methods were conducted 2 m from an experimental hut inside the SFS to simulate an outdoor peridomestic setting (Fig. 1a and b). Experiments were conducted between 06:30 and 09:30 every day to reflect the natural *Ae. aegypti* biting time. The experiment started when the volunteer sat on the chair and simultaneously 100 mosquitoes (from four holding cages, each with 25 mosquitoes) per chamber were released on a signal from the team supervisor. HLC and MET collections were done continuously for 50 min with a 10-min break at the end of each hour [31]. During the break, the MET was switched off to allow collection of mosquitoes that had been trapped between stainless steel wires or had fallen on the ground due to electrocution. Because opening and closing the door for the volunteer to take a break outside the SFS would cause mosquitoes escape, during this time, volunteers remained inside with their trousers unfolded to restrict mosquitoes' bites. For the same reason, the BGS trap was emptied after 3 h. Collected mosquitoes were kept in waxed paper cups with net lids, labeled with the time, date and method of collection and then transported to the insectary for counting and recording. After each experimental day, a thorough search within SFS chamber was conducted, and all mosquitoes that were not recaptured were aspirated using a Prokopack to avoid contamination of the replicates.

Under the “choice assay" or “competitive experimental assay” similar experimental procedures and trapping types as above were employed, with the exception that in addition to a trapping type assigned to a particular experimental day, a volunteer conducting HLC was added and positioned 10 m away from the FTPE in each chamber (Fig. 1b). This was to simulate the competition for mosquitoes that could happen when these collection methods are used in the field. This setup also enabled determination of whether mosquitoes in the presence of transfluthrin were diverted to the unprotected volunteer performing HLC and detection of whether there was an increase in biting compared to the control.

### Sample size

Sample size calculations were performed using simulation-based power analysis [32] in R statistical software version 3.3 (http://www.r-project.org) with a significance level of 0.05 for rejecting the null hypothesis. The power to predict the 15% difference in mosquito landings among the HLC, MET and BGS traps was estimated as the proportion of the 1000 simulated data sets in which the null hypothesis was rejected when the generalized linear mixed model (GLMM) was run. Inter-observational variance among daily experiments (0.5) was adapted from a previous study conducted in the semi-field. With our experimental design and a predicted 60% recapture rate of released mosquitoes by HLC in the control (reference method), there was 98% power to detect a difference.

### Data analysis

Analyses of data were carried out in Stata 13 (StataCorp). Hourly data were collapsed to give the total of mosquitoes caught per trap so that data for all three methods were comparable (Additional file 1). Data analyses for the choice and no-choice experiments were performed separately. The mean percentage of recapture and confidence intervals (CI) were calculated for each collection method in the treatment and control in the no-choice and choice scenarios. The overall arithmetic mean PE and 95% CI for the experiment were calculated from the daily PE, which was measured by comparing the human landing rate on a volunteer with the intervention to the negative control using the following formula:

$${\text{Protective efficacy}}\, = \,\left[ {{{\left( {C{-}T} \right)} \mathord{\left/ {\vphantom {{\left( {C{-}T} \right)} C}} \right. \kern-\nulldelimiterspace} C}} \right]\, \times \,{1}00\% ,$$

where *C* stands for the number of mosquitoes landing in the control and *T* is the number of mosquitoes landing in the treatment.

Three GLMMs with a binomial distribution with the logit link were used to determine the following: (i) the ability of the traps to measure the protection conferred by the FTPE in a no-choice experiment, (ii) the ability of the traps to measure the protection provided by the FTPE in a choice experiment and (iii) the difference in the proportion of recaptured mosquitoes by HLC in position 2 (competitor HLC) when HLC was used. This allowed ascertaining whether there was any diversion from the HLC in position 1 to the competitor HLC caused by the transfluthrin. Diversion was defined as the movement of mosquitoes from the HLC in position 1 to the HLC in position 2. The diversion of mosquitoes was evaluated by the odds of recapturing a mosquito in the competitor HLC (position 2) relative to position 1 for HLC only.

In all models, the independent variables included as fixed categorical effects were collection method, treatment (FTPE or control), temperature and humidity, with experimental day as a random effect. An interaction term between treatment and collection methods was introduced to determine if the reduction in landing caused by the VP was measured differently by collection method.

Relative trap efficacy, that is, the ratio of mosquitoes recaptured in each trap relative to HLC, was calculated for the choice and no-choice experiments for both the transfluthrin and the control arms.

## Results

### Traps and HLC measure similar protective effect of transfluthrin in the no-choice test

When HLC, MET or BGS was used to collect mosquitoes with FTPE placed at 3 m on both sides of the collection method, approximately 22% of the mosquitoes were collected (MET: odds ratio [OR] 0.82 [95% CI: 0.69–1.14], *P* = 0.245; BGS: OR: 0.89 [95% CI: 0.64–1.24], *P* = 0.490; Table 1). In the control, similar proportions of mosquitoes (> 60%) were recaptured using HLC and BGS traps, although the MET showed lower trapping efficacy relative to HLC (MET: OR: 0.34 [0.25–0.46], *P* < 0.001; BGS: OR: 0.61 [95% CI: 0.45–0.83], *P* = 0.002; Table 1). This meant that while all collection methods measured that the FTPEs reduced mosquito landings, the reduction measured with the MET (OR: 0.29 [95% CI: 0.24–0.37], *P* < 0.001) was less than that measured with the BGS trap (OR: 0.18 [95% CI: 0.18–0.23], *P* < 0.001) and with HLC (OR: 0.12 [95% CI: 0.09–0.15], *P* < 0.001). The results of the interaction between collection method and treatment indicated that these differences were significant for MET (OR: 2.4 [95% CI: 1.75–3.03], *P* < 0.001) and for BGS (OR: 1.45 [95% CI: 1.05–1.98], *P* = 0.022).

**Table 1:** Relative trapping efficacy of human landing catch (HLC), mosquito-electrocuting traps (METs) and BG-Sentinel (BGS) traps evaluated individually in a no-choice test and in competition with HLC in a choice test, in the presence of two freestanding transfluthrin passive emanators (treatment) and with no transfluthrin (control)

	Transfluthrin						Control					
	Total recapture (% released)	“Trap” recapture position 1 (% recaptured)	HLC recapture position 2 (% recaptured)	Relative trapping efficacy^a^	OR (95% CI)^b^	*P*-value	Total recapture (% released)	“Trap” recapture position 1 (% recaptured)	HLC recapture position 2 (% recaptured)	Relative trapping efficacy^a^	OR (95% CI)^b^	*P*-value
No-choice												
HLC		192 (24)	–	1	1	–		571 (71)	–	1	1	–
MET		163 (20)	–	0.85	0.82 (0.69–1.14)	0.245		372 (47)	–	0.63	0.34 (0.25–0.46)	< 0.001
BGS		174 (22)	–	0.91	0.89 (0.64–1.24)	0.490		487 (61)	–	0.85	0.61 (0.45–0.83)	0.002
Choice												
HLC + HLC	402 (50)	208 (26)	194 (24)	1	1	–	711 (89)	335 (42)	376 (47)	1	1	–
MET + HLC	232 (29)	22 (2)	210 (26)	0.11	0.07 (0.04–0.13)	< 0.001	480 (60)	96 (12)	384 (48)	0.29	0.18 (0.13–0.25)	< 0.001
BGS + HLC	307 (38)	38 (5)	269 (34)	0.18	0.05 (0.02–0.13)	< 0.001	618 (77)	53 (7)	565 (71)	0.16	0.09 (0.05–0.15)	< 0.001

The average proportion of released mosquitoes that was recaptured in the transfluthrin and control arm is presented as well as the total number of mosquitoes recaptured by each method out of 800 *Aedes aegypti* mosquitoes released over eight replicates

^a^Relative trapping efficacy of METs and BGS traps compared to HLC in no-choice and choice tests in both the treatment and the control arms (both in position 1)

^b^The odds ratio (OR) estimates were derived from Stata output adjusted for temperature and humidity and presented with a 95% confidence interval

When the protection provided by the FTPE was calculated using the PE, which is not adjusted for other sources of variation (such as location, day and volunteer), all the collection methods measured similar PE of approximately 60% (MET: 55% [95% CI: 48–63], HLC: 66% [95% CI: 50–82]; BGS: 64% [95% CI: 54–73]; Table 2).

**Table 2:** Protective efficacy of freestanding transfluthrin passive emanators (FTPEs) measured using human landing catch (HLC), mosquito-electrocuting traps (METs) and BG-Sentinel (BGS) traps evaluated individually in a no-choice test and in competition with HLC in a choice test in the presence of two FTPEs (treatment) and with no transfluthrin (control)

Trap Combination	Position 1					Position 2				
	% recapture Control (95% CI)	% recapture Treatment (95% CI)	%Protective efficacy (95% CI)	OR (95% CI)^a^ treatment relative to the control	*P*-value	% recapture Control (95% CI)	% recapture Treatment (95% CI)	Protective efficacy (95% CI)	OR (95% CI)^a^ treatment relative to the control	*P*-value
No-choice										
HLC	71 (63–80)	24 (14–34)	66 (50–82)	0.12 (0.09–0.15)	< 0.0001					
MET	47 (41–52)	20 (18–23)	55 (48–63)	0.29 (0.24–0.37)	< 0.0001					
BGS	61 (53–68)	22 (17–27)	64 (54–73)	0.18 (0.15–0.23)	< 0.0001					
Choice										
HLC + HLC	42 (39–45)	26 (22–30)	37 (25–50)	0.49 (0.39–0.60)	< 0.0001	47 (43–51)	24 (14–34)	49 (30–68)	0.35 (0.28–0.43)	< 0.0001
MET + HLC	12 (7–17)	2 (1–4)	76 (61–92)	0.20 (0.13–0.33)	< 0.0001	48 (42–54)	26 (21–32)	44 (31–57)	0.38 (0.31–0.47)	< 0.0001
BGS + HLC	7 (4–9)	5 (0–10)	0 (0–100)	0.70 (0.45–1.08)	0.105	71 (65–76)	34 (26–41)	52 (40–64)	0.21 (0.17–0.26)	< 0.0001

The percentage of released mosquitoes recaptured by each method is presented out of 800 *Aedes aegypti* mosquitoes released over eight replicates. The proportion recaptured is the arithmetic mean recaptured out of the total released

^**a**^The odds ratio (OR) estimates were derived from Stata output of treatment relative to control from mosquitoes recaptured in position 1 in the presence (choice) and absence (no-choice) of additional HLC conducted in position 2, adjusted for temperature and humidity and presented with a 95% confidence interval

### Traps and HLC did not measure a similar protective effect of transfluthrin in the choice test

In choice tests, the combined number of mosquitoes recaptured by both collection methods was higher than in a single trap in the no-choice tests; however, the presence of a second human substantially reduced mosquito numbers caught in all of the collection methods at position 1. In the treatment, 208 (52%), 22 (9%) and 38 (12%) mosquitoes were recaptured by HLC, MET and BGS traps, respectively; in the control, the corresponding numbers were HLC: 335 (47%), MET: 96 (20%) and BGS: 53 (9%; Table 1). In the treatment, the model showed that HLC in position 1 recaptured a significantly higher proportion of *Aedes* mosquitoes than either the MET or BGS trap: MET [OR: 0.07 (95% CI: 0.04–0.13), *P* < 0.0001]; BGS [OR: 0.05 (95% CI: 0.02–0.13), *P* < 0.0001] (Table 1). A similar trend was observed when the trap performances were compared in the control with MET: [OR: 0.18 (95% CI: 0.13–0.25), *P* < 0.0001] and BGS: [OR: 0.09 (95% CI: 0.05–0.15), *P* < 0.0001] (Table 1). When HLC is a reference, the model showed a significant interaction between HLC and MET but not with HLC and BGS. This indicated that the reduction in landing caused by the FTPE as measured by HLC and the BGS trap was not significantly different (OR: 1.44 [95% CI: 0.89–2.33], *P* = 0.13) but that of the MET measured higher protection than HLC (OR: 0.42 [95% CI: 0.25–0.71], *P* < 0.001). Significant reduction in the odds of landing of *Ae. aegypti* was observed using HLC (OR: 0.49 [95% CI: 0.39–0.60], *P* < 0.001), while MET measured a slightly higher protection (OR: 0.20 [95% CI: 0.13–0.33], *P* < 0.001); protection could not be measured for the BGS trap as the confidence interval crossed 1 (OR: 0.70 [95% CI: 0.45–1.08], *P* < 0.105; Table 2).

When assessing the impact of the FTPE using PE basic formula, HLC in position 1 measured a PE of 37.2% (95% CI: 25.0–49.5); the MET overestimated PE at 75% (95% CI: 60.5–91.5) while the PE estimate for BGS was not measurable because of low attraction to the BGS trap (PE 0% [95% CI: 0–99.5]; Table 2).

The proportion of recaptured mosquitoes for the second HLC sitting in position 2, located 10 m away in the treatment, ranged from 24 to 34%, whereas in the control the proportion of recaptured mosquitoes was 47% to 71% for all traps used. This means that FTPEs provided a consistent protection of about 50% to the second HLC sitting in position 2 independent of which mosquito collection method, HLC, MET or BGS, was used in position 1 (Table 2).

### No evidence of mosquito diversion from HLC position 1 to HLC in position 2 at 10 m in the presence of transfluthrin

Mosquito diversion was assessed from the relative proportion caught by HLC in position 1, 3 m from the FTPE, and position 2, 10 m from the FTPE, in the treatment. There was no diversion of mosquitoes from the HLC in position 1 to the HLC in position 2 in the presence of FTPE (OR: 0.87 [95% CI: 0.66–1.15], *P* = 0.324; Table 3). In both positions 1 and 2, HLC captured similar proportions of mosquitoes in the presence of FTPE and control, at a ratio of approximately 1:1 (Table 2).

**Table 3:** Diversion of mosquitoes from human landing catch (HLC), mosquito-electrocuting traps (METs) and BG-Sentinel (BGS) traps in position 1, located 3 m from freestanding transfluthrin passive emanators (FTPEs) or control to a person conducting HLC in position 2, 10 m from FTPEs or control

Trap combination	Transfluthrin					Control				
	“Trap” recapture position 1	HLC recapture position 2	Recapture in position 2 relative to position 1	OR (95% CI)^a^ position 2 relative to position 1	*P*-value	“Trap” recapture position 1	HLC recapture position 2	Recapture in position 2 relative to position 1	OR (95% CI)^a^ position 2 relative to position 1	*P*-value
HLC + HLC	208	194	0.93	0.87 (0.66–1.15)	0.324	335	376	1.12	1.26 (1.02–1.55)	0.030
MET + HLC	22	210	9.55	91.1 (49.0–170.0)	< 0.0001	96	384	4.00	16.0 (11.66–22.0)	< 0.0001
BGS + HLC	38	269	7.08	50.1 (31.0–81.0)	< 0.0001	53	565	10.66	113.6 (76.3–169.2)	< 0.0001

The percentage of released mosquitoes recaptured by each method is presented out of 800 *Aedes aegypti* mosquitoes released over eight replicates. The proportion recaptured is the arithmetic mean recaptured out of the total released

^**a**^The odds ratio (OR) estimates were derived from Stata output of position 2 relative to position 1 in the presence of transfluthrin or control, adjusted for temperature, humidity, compartment and volunteers and presented with 95% confidence interval

### Evidence that the presence of a human at 10 m attracts all mosquitoes away from BGS trap and MET

In the presence of either FTPE or control, the relative recapture by the HLC in position 2 was higher in the presence of a BGS trap or MET (Table 4). In the control, it was observed that the HLC in position 2 caught 1.5 times more mosquitoes (565; 91%) in the presence of the BGS trap than in the presence of the HLC (376 [53%]; OR: 3.37 [95% CI: 2.35–4.85], *P* < 0.0001). A similar but less pronounced trend was observed in the presence of transfluthrin, with the HLC in position 2 receiving 1.39 times more mosquitoes than if a second HLC was being conducted (269 [88%] with BGS and 194 [48%] with HLC; OR = 1.63 [95% CI: 0.79–3.34], *P* = 0.184; Table 4). With MET, more mosquitoes were recaptured by HLC than by the MET, but the number caught by HLC did not increase in either the control (OR: 1.06 [95% CI: 0.85–1.33], *P* = 0.593) or the treatment (OR: 1.15 [95% CI: 0.72–1.84], *P* = 0.547) arm. Because recaptures did not increase using HLC, the lower proportion of mosquitoes recaptured by the MET is likely due to lower trapping efficiency, whereas humans were clearly more attractive than the BGS trap.

**Table 4:** Relative recapture of competing human landing catch (HLC) in the presence of HLC, mosquito-electrocuting traps (METs) and BG-Sentinel (BGS) traps in a choice test in the presence of two freestanding transfluthrin passive emanators (treatment) and with no transfluthrin (control)

Trap combination	Transfluthrin				Control			
	HLC recapture position 2 (% of recapture)	Recapture in position 2 relative to HLC in position 1^a^	OR (95% CI)^b^	*P*-value	HLC recapture position 2 (% of recapture)	Relative recapture in position 2 relative to position 1^a^	OR (95% CI)^b^	*P*-value
HLC + HLC	194 (48)	1	1	–	376 (53)	1	1	
MET + HLC	210 (91)	1.08	1.15 (0.72–1.84)	0.547	384 (80)	1.02	1.06 (0.85–1.33)	0.593
BGS + HLC	269 (88)	1.39	1.63 (0.79–3.34)	0.184	565 (91)	1.50	3.37 (2.35–4.85)	< 0.0001

The average proportion of released mosquitoes that was recaptured by each method by HLC in position 2 is presented out of 800 *A. aegypti* mosquitoes released over eight replicates

^a^Relative recapture in HLC position 2 when HLC is conducted in position 1 compared to the MET and BGS trap in position 1 in both the treatment and the control arms

^b^The odds ratio (OR) estimates were derived from Stata output adjusted for temperature and humidity and presented with a 95% confidence interval

### Relative trap efficiency in the absence of transfluthrin and competitor

In the experiments with the no-choice setup, in the control, it was possible to calculate the relative trapping efficiencies expected if the traps were used for monitoring of human exposure to the *Ae. aegypti* and if no other kairomones were present. Both the MET and the BGS trap collected fewer mosquitoes than the HLC. The recapture measured with the MET (OR: 0.34 [95% CI: 0.25–0.46], *P* < 0.001) and the BGS trap (OR: 0.61 [95% CI: 0.45–0.83], *P* = 0.002) differed from that of the HLC. The MET collected approximately 37% fewer mosquitoes, and the BGS trap collected approximately 15% fewer mosquitoes (Table 1). Based on these data, for mosquito sampling as a measure of relative human exposure to *Ae. aegypti*, the BGS trap and the MET can be used with correction factors of 1.6 and 1.2, respectively.

## Discussion

Outdoor vector control tools such as spatial repellents, including VP, promise to be an important addition to the vector control toolbox because they protect multiple users within a defined space. The current study compared the efficacies of the gold standard, HLC, and two exposure-free mosquito-collection methods, MET and BGS traps, in estimating the protective efficacy of the VP. The protective efficacy measured by each trapping method was evaluated either independently or in the presence of an additional HLC to simulate competition between blood hosts and its impact on mosquito behavior [22].

### Traps and HLC measure a similar protective effect of transfluthrin in the no-choice test

This study demonstrated that in the absence of a HLC competitor, similar protective efficacy of VP was measured by the BGS trap, MET and HLC using the basic formula based on unadjusted mean mosquito landings. However, in the statistical model, a significant interaction between trap and treatment showed that MET and HLC measured the protective effect of the transfluthrin differently. The differences between the model estimates for the OR and the basic formula for PE may be explained by the fact that the model is adjusted to other variables. However, this difference between HLC/BGS and MET, with MET being 10% lower than the others, is too small for the basic PE formula to detect. Therefore, it can be inferred that field experiments to evaluate VP using exposure-free methods of *Ae. aegypti* collection are possible provided that the experiments are sufficiently well powered and are designed to ensure independence of observations without the bias of alternative host cues. Because it is not ethical to measure PE in the viral-endemic area using HLC, this small degree of error in estimating PE is acceptable. Furthermore, in field experiments, the incidence rate ratio will be calculated from mosquito count data adjusted for sources of variation, allowing estimation of the adjusted protective efficacy using IRR [33]. In the current experiments, a binomial distribution was used because a known number of released mosquitoes is known when conducting experiment in the semi field system. Independence of observations is an essential consideration in the design of experiments, and field trials using METs or BGS traps, as a proxy for HLC, must be conducted in locations away from competing sources of attraction. This result was encouraging because the use of METs or BGS traps would allow safer field evaluation of VP in areas of active arbovirus transmission where HLC is not possible, although it must be understood that measures of protection are not exact because of the limitations of the traps used.

In the control, MET collected approximately half the number of mosquitoes caught by HLC, and the BGS trap about 15% fewer. Similar results have been seen repeatedly in other studies with different traps because odor baited traps generally provide some but not the complete suite of host cues required to maximize mosquito attraction. One exception is the host decoy trap (HDT), which provides whole-host odor, visual cues and heat [19]. Even so, the number of *Anopheles* mosquitoes caught by HLC was higher than that with HDT in southeast Asia [34] and compared to other human-baited traps, such as human double-net traps in Laos [18] and the MET in Tanzania [35]. A study conducted in Ecuador showed that the mean *Ae. aegypti* collected when using MET or BGS in the field was equal [26], which contrasts with the current findings. This difference may be due to the closed SFS environment in which the traps were evaluated for the current study or to the low density of *Ae. aegypti* captured in the Colombian study. Furthermore, in the Colombian study *Culex quinquefasciatus* was highly abundant and the MET collected fewer of this species than did the BGS trap [26].

The presence of host cues is an important consideration in testing repellents because it is known that molecules such as N, N-diethyl-3-methylbenzamide (DEET) interact with host odor receptors [36]. As the MET and HLC methods use humans as bait, we would expect similar proportions of recaptured mosquitoes. The differences in catch size may be explained by the fact that day-active *Ae. aegypti* use visual cues to locate their host [37]. It is therefore possible that they are more aware of the electric grid [38] or are unable to pick up as many short-range cues such as thermal and water vapor cues [39, 40]. Nonetheless, this finding warrants further comparison of BGS traps and METs under field conditions to confirm these promising SFS findings for monitoring *Ae. aegypti* in Tanzania. The advantages of using MET or BGS trap mosquito collection methods as an alternative to HLC for monitoring human exposure to *Ae. aegypti* include: it removes variation caused by individual skill and motivation to collect mosquitoes, it is far safer, and does not require extensive user training.

### Traps and HLC did not measure similar protective efficacy of transfluthrin in the choice test

The presence of a second competitor HLC in the SFS strongly affected the estimated personal PE of the FTPE. It is difficult to interpret the results because very few mosquitoes were caught in the MET or BGS trap when there was a human competitor and therefore the power to measure the difference in treatment and control was very low. This result showed that human competitors could significantly affect the traps’ collection ability. These experiments were conducted in the SFS, where the number of mosquitoes is limited to those released, and it may therefore be possible to increase power to detect the difference by using more mosquitoes. Because space and host options for the mosquitoes are also limited, it would be useful to confirm whether these results would be reflected in a field trial. However, there are ethical concerns in doing HLC in the field except in an area with no known arbovirus transmission.

A significant interaction between trap and treatment showed that METs, HLC and BGS traps measure the effect of transfluthrin differently. This was consistent even when the basic formula for PE was used to assess the efficacy of the collection methods in evaluating VP. The presence of a competitor HLC reduced the precision of METs and BGS traps in measuring PE. However, this may reflect the true PE that could be measured in the field, where the possibility of finding someone in isolation is very small. The average PE was 62% in the no-choice experiments, which is consistent with other evaluations of FTPEs [29]. However, in the choice tests, BGS traps measured a reduced PE and increased PE was measured by METs. This is explained by the presence of a second HLC, which introduces other cues, causing variability in the data. It is known that mosquitoes orient to carbon dioxide (CO_2_) from over 20 m [41] and select between hosts at distances of approximately 15 m [42]. Consequently, it is recommended that topical repellents be tested with individuals over 20 m apart [43] in no-choice tests [44] to ensure independence of observations. The current data add weight to this recommendation. It is consistent with observations that household mosquito densities are correlated with the number of occupants [45]. In addition, other studies of transfluthrin PE in semi-field systems demonstrated that the addition of a CO_2_-baited Suna trap reduced transfluthrin PE and that the trap did not perform well in the presence of a human [46]. This is consistent with the current findings that protective efficacy of transfluthrin was lower, but not significantly so, in the presence of a second competitor HLC; BGS traps and METs collected substantially fewer mosquitoes.

### No evidence of mosquito diversion from a protected individual to a second individual at 10 m in the presence of transfluthrin

Spatial repellents, including VPs, are an important addition to the vector control toolbox because they protect multiple users within a defined space [47]. This study demonstrated that the presence of FTPEs in all of the experimental configurations (HLC, MET and BGS traps) reduced the number of collected mosquitoes. The competitor HLC, located 10 m from the FTPEs, also demonstrated approximately 50% PE. This is consistent with another study conducted against *Anopheles arabiensis* (*An. arabiensis*) in Tanzania and *An. harrisoni* and *An. minimus* in Thailand where the overall protective efficacy of 50% extended 5 m and 10 m in an outdoor setting [13, 48]. However, in the Thailand study both the treatment and the control were in the same compartment. Considering the mechanism of action of transfluthrin, with this experimental design the PE observed might be underestimated. Thus, independence of the treatment arms is very important during the evaluation of a volatile pyrethroid such as transfluthrin.

This study showed that VPs act on mosquitoes over distances of several meters with a non-contact (spatial) mode of action [49]. From a public health perspective, this is a useful robust characteristic of VPs used as spatial repellents because they can protect multiple users with no need for daily compliance, unlike topical repellents, which suffer from diversion of users to non-users [50] and extremely low daily compliance among users in endemic countries [51], travelers [52] and military populations [53]. Further testing of the usefulness of METs for the evaluation of topical repellents that act over distances of just a few centimeters [54] is required to validate METs for evaluation of other bite prevention interventions, such as topical repellents and insecticide-treated clothing.

While there is some evidence that VPs can cause an increase in mosquito bites among non-repellent using households in villages with incomplete coverage of VP [55], it has also been observed that when applied at large scale, transfluthrin VP can reduce malaria [56]. This is because transfluthrin has multiple modes of action. It can cause rapid knockdown and kill [57] and feeding inhibition up to 12 h post-exposure, referred to as “disarming” [58], as well as causing landing reduction, which is important when considering the use of this intervention at scale for public health [59]. While diversion was not observed in this study, we cannot rule out the possibility of diversion occurring in other settings where an individual may be positioned outside the reach of the protective radius of transfluthrin.

### Evidence that humans attract the majority of mosquitoes at 10 m in the presence of BGS traps

This study also observed that humans positioned 10 m away from a BGS trap received all the mosquito landings, similar to if they had been positioned alone. While the presence of transfluthrin did continue to protect the HLC participant in the presence of the BGS, in the control arm, mosquito landings substantially increased. This is unsurprising, because mosquito sensitivity to skin odors has been shown to increase at least fivefold immediately following a brief encounter with a filament of CO_2_ [60]. This mechanism may also explain the findings of a similar study in an SFS in Kenya, where transfluthrin showed lower PE in the presence of an odor-baited Suna trap than when used without the trap [46]. However, the authors point out that the differing ambient temperatures, which may affect release rates of VPs, may have confounded their data.

The same finding was observed in push-pull evaluations in Tanzania [61] in which increasing odor-baited trap density around houses increased landings on people conducting HLC while moving traps farther away was protective [61]. Therefore, the location of traps with CO_2_ for *Ae. aegypti* surveillance should be carefully considered in areas of active arbovirus transmission to ensure that householders where traps are located do not experience increased bites. This finding has also be seen in Tanzania [62], where odor-baited traps lured large numbers of mosquitoes from a distance but could not compete with humans at short range and actually resulted in increased landings for those sitting close to odor-baited traps. This causes difficulties: if the traps are moved out of peridomestic areas, they will likely no longer be able to measure the impact of peridomestic interventions such as VPs. So while odor-baited traps with CO_2_ are being considered because their use will be safer for the HLC technicians, there may be unwanted side effects for community members.

### Other considerations for repellent evaluations

In our study, the paired HLC captured similar proportions of mosquitoes in the absence of VPs, with a ratio of approximately 1:1. The participation of highly skilled technicians collecting over 3 h allowed equivalent estimation of mosquito landings although the studies were performed at different times. This highlights the importance of training and supervision of staff involved in the conduct of entomological evaluations. The technical staff were highly motivated to perform the test accurately following discussion of the importance of the study and their role in the generation of accurate data [63]. Also, it is important to highlight that during the evaluation of the spatial repellent the number of mosquitoes collected using odor-baited trap may be reduced. This may over-estimate the efficacy of spatial repellents when odor-baited traps are used. Baseline information before the implementation of the trial must be conducted.

### Study limitations

First, during collection the BGS trap ran continuously for 3 h while each hour a 10-min break was provided for those conducting MET testing or HLC to stretch and to collect mosquitoes from the MET. Thus, the total sampling time for the BGS trap was 3 h, whereas it was 2 h 30 min for both the HLC and MET. Therefore, the number of mosquitoes caught by the BGS trap may be overestimated. Second, the volunteers observed that mosquitoes electrocuted by the MET occasionally recovered and flew away, which may contribute to a lower estimate of the mosquito landing rate. This study used 680 V generated by the MET, but for those experiments conducted in the SFS that do not need mosquito samples after electrocution, higher voltage may be used. Third, the experiments were conducted in the semi-field system using laboratory-reared mosquitoes. Although the mosquitoes were recently colonized, it is possible that these results may not represent what would happen in a real-world situation with wild mosquitoes. In addition, the results may not be generalizable to all mosquito species. While the data were consistent with those from other experiments using a similar dose of transfluthrin, the relative efficacy of the BGS trap and the MET to estimate PE may vary according to transfluthrin concentration. Further experiments with varying doses of transfluthrin conducted in multiple settings would be useful to strengthen the findings of this study.

## Conclusions

HLC, METs and BGS traps measured a consistent 60% PE of transfluthrin emanator in isolation from competing host cues, the PE estimated by each method was variable in the presence of an HLC competitor. Therefore, measurement of the PE, that is, reduction in landings of mosquitoes caused by VP spatial repellents, is possible using HLC, METs or BGS traps in no-choice tests. While HLC is probably a better measure of the PE offered by the volatile pyrethroid because the whole suite of medium- and short-range host cues is available to host-seeking mosquitoes, ethical concerns in arbovirus-endemic areas restrict its use in the field. This study suggests that estimation of the PE of VPs or other spatially acting compounds against anthropophilic mosquitoes such as *Ae. aegypti* could be evaluated in the field using either METs or BGS traps provided that independence of observations can be met. Findings also indicate that transfluthrin can protect multiple people in the peridomestic area and that using a BGS trap close to people may increase their exposure to host-seeking mosquitoes that are attracted by CO_2_ at long range and then select humans at short range. This study needs to be repeated in other sites to confirm the findings.

## Supplementary Information

**Additional file 1.** Data from the semi-field comparison of HLC, METs and BGS traps with competitor HLC and HLC, METs and BGS traps without competitor HLC in the presence and absence of spatial repellent for catching *Ae. aegypt*i mosquitoes.

## Data Availability

All data generated and analyzed for this research article are included as Additional file 1.

## Abbreviations

HLC: Human landing catch
CI: Confidence interval
EC: Emulsified concentrate
VP: Volatile pyrethroid
MET: Mosquito-electrocuting trap
BGS: BG-Sentinel
PE: Protective efficacy
HLR: Human landing rate
SFS: Semi-field system
CO_2_: Carbon dioxide
CDC: Centers for Disease Control
DC: Direct current
DEET: *N*, *N*-diethyl-3-methylbenzamide
HDT: Host decoy trap
