# Supplementary figures and images for: Semi-field evaluation of the exposure-free mosquito electrocuting trap and BG-Sentinel trap as an alternative to the human landing catch for measuring the efficacy of transfluthrin emanators against Aedes aegypti
Source: Parasit Vectors. 2021 May 20;14:265. doi: 10.1186/s13071-021-04754-x (PMC8138975; doi:10.1186/s13071-021-04754-x)

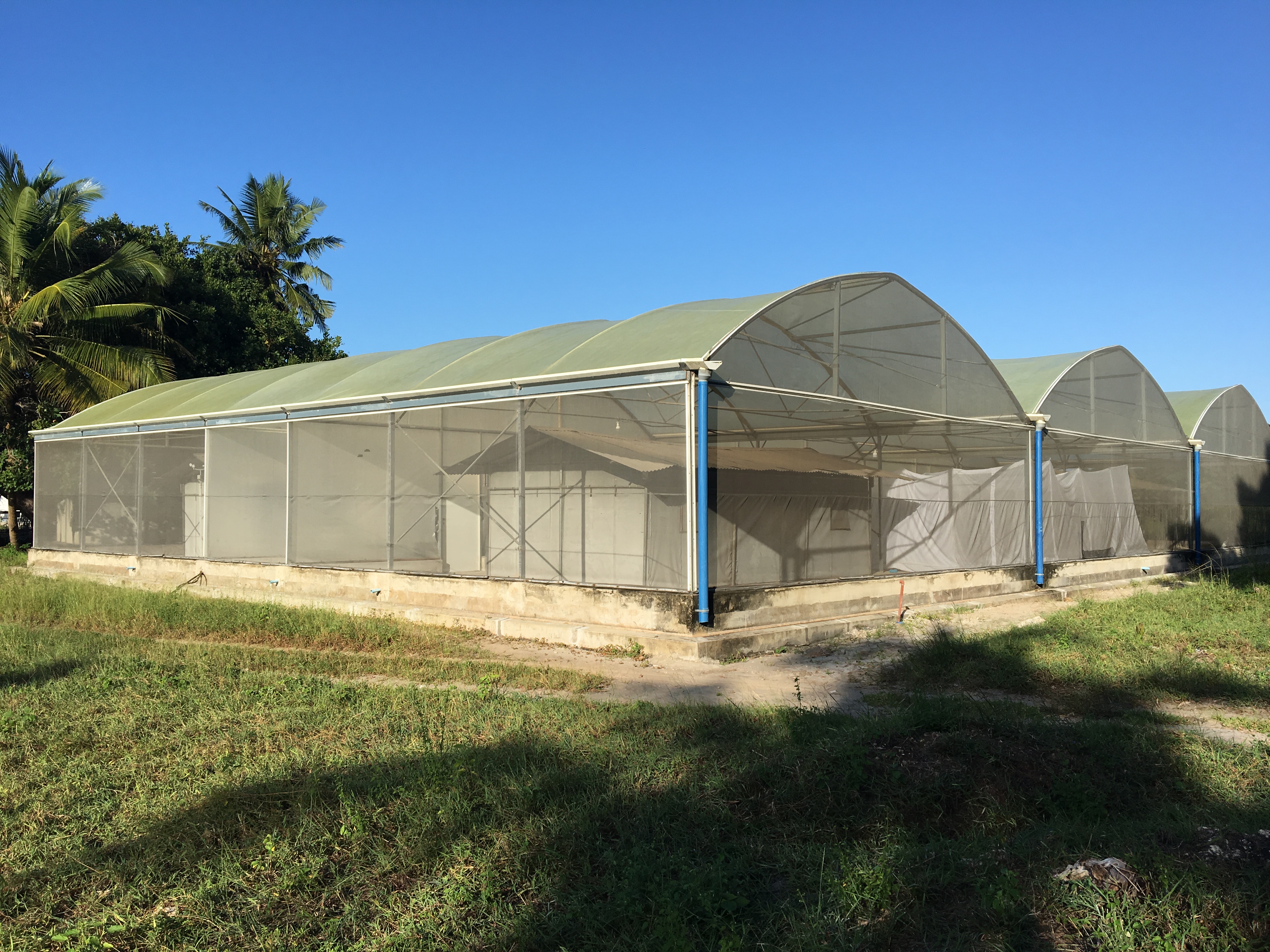

Supplement: Supplementary file 1 — Additional file 1. Data from the semi-field comparison of HLC, METs and BGS traps with competitor HLC and HLC, METs and BGS traps without competitor HLC in the presence and absence of spatial repellent for catching Ae. aegypti mosquitoes. [file 13071_2021_4754_MOESM1_ESM.jpg]
